# Supplementary material for: Sedative Properties of Dexmedetomidine Are Mediated Independently from Native Thalamic Hyperpolarization-Activated Cyclic Nucleotide-Gated Channel Function at Clinically Relevant Concentrations
Source: Int J Mol Sci. 2022 Dec 28;24(1):519. doi: 10.3390/ijms24010519 (PMC9820684; doi:10.3390/ijms24010519)
Supplement: Supplementary file 1 [file ijms-24-00519-s001.zip › ijms-2087452-supplementary.pdf]

Supplementary Material

Sedative properties of dexmedetomidine are mediated independently from native mice thalamic hyperpolarization-activated, cyclic nucleotide gated channel function at clinically relevant concentrations

Supplementary Table S1. Effects of dexmedetomidine on the resting membrane potential at different concentration groups (absolute values) after 30 minutes and 45 minutes of DEX wash in. There were no significant differences between control, 30 minutes, and 45 minutes of DEX (1, 10, and 100 μM) exposure on the resting membrane potential. For multiple comparisons of repeated measures, Friedman test was used.

	**resting membrane potential [mV]**							
	**control**		**DEX 30 min**		**DEX 45 min**			
**DEX [μM]**	Median	IQR	Median	IQR	Median	IQR	n	*p*
**1**	−58.55	−61.12 – −55.77	−60.03	−62.78 – −58.41	−58.85	−61.86 – −56.85	11	0.219
**10**	−58.17	−60.23 – −57.13	−58.04	−61.44 – -56.14	−57.60	−61.25 – −56.17	10	0.368
**100**	−57.48	−58.47 – −56.91	−58.54	−60.5 – -56.97	−58.73	−59.68 – −57.11	10	0.067

Supplementary Table S2. Effects of dexmedetomidine (45 minutes) on the resting membrane potential at different concentration groups (absolute values). Wilcoxon-matched pairs signed rank test for statistical comparisons.

	**resting membrane potential [mV]**					
	**control**		**DEX**			
**DEX [μM]**	Median	IQR	Median	IQR	n	*p*
**1**	−58.55	−61.12 – −55.77	−58.85	−61.86 – −56.85	11	0.465
**10**	−58.17	−60.23 – −57.13	−57.60	−61.25 – −56.17	10	0.846
**100**	−57.48	−58.47 – −56.91	−58.73	−59.68 – −57.11	10	0.106

Supplementary Table S3. Effects of dexmedetomidine (45 minutes) on the input resistance at different concentration groups (absolute values). Wilcoxon-matched pairs signed rank test for statistical comparisons.

	**input resistance [MΩ]**					
	**control**		**DEX**			
**DEX [µM]**	Median	IQR	Median	IQR	n	*p*
**1**	290.3	235.4–343.4	317.4	260.8–337.8	11	0.044
**10**	362.7	311.5–386.3	393.1	362.5–420.7	10	0.006
**100**	339.4	261.9–413.9	424.5	369.1–493.3	10	0.002

Supplementary Table S4. Effects of dexmedetomidine (45 minutes) on the action potential threshold at different concentration groups (absolute values). Wilcoxon-matched pairs signed rank test for statistical comparisons.

	**action potential threshold [mV]**					
	**control**		**DEX**			
**DEX [µM]**	Median	IQR	Median	IQR	n	*p*
**1**	−38.22	−39.98 – −37.45	−40.28	−41.69 – −36.84	11	0.102
**10**	−38.64	−39.50 – −36.49	−39.38	−41.16 – −37.55)	10	0.232
**100**	−39.80	−40.97 – −39.27	−38.16	−38.98 – −33.25	10	0.002

Supplementary Table S5. Effects of dexmedetomidine (45 minutes) on the action potential at different concentration groups (absolute values). Wilcoxon-matched pairs signed rank test for statistical comparisons.

	action potential frequency [Hz]					
	control		DEX			
**DEX [μM]**	Median	IQR	Median	IQR	n	*p*
**1**	39.67	36.46–39.84	41.96	36.59–45.19	11	0.054
**10**	42.48	34.56–45.57	41.26	35.01–46.52	10	>0.999
**100**	42.08	39.75–45.30	27.44	21.61–33.66	10	0.002

Supplementary Table S6. Effects of dexmedetomidine (45 minutes) on $I_{h\ maximal}$ (recorded at -133 mV) at different concentration groups (absolute values). Wilcoxon-matched pairs signed rank test for statistical comparisons.

	$I_{h\ maximal}$ [pA]					
	control		DEX			
**DEX [μM]**	Median	IQR	Median	IQR	n	*p*
**1**	516	479–983	495	378–838	11	0.042
**10**	534	321–719	434	305–663	10	0.006
**100**	605	418–860	429	321–500	10	0.004

Supplementary Table S7. Effects of dexmedetomidine (45 minutes) on the half-maximal activation potential of HCN channels at different concentration groups (absolute values). Wilcoxon-matched pairs signed rank test for statistical comparisons.

	**half-maximal activation potential [mV]**					
	**control**		**DEX**			
**DEX [µM]**	Median	IQR	Median	IQR	n	*p*
**1**	−86.23	−87.32 – −84.81	−86.64	−87.63 – −84.65	11	0.898
**10**	−87.42	−88.70 – −86.22	−86.89	−88.47 – −85.29	10	0.695
**100**	−85.88	−88.15 – −83.99	−91.54	−93.22 – −88.07	10	0.004

Supplementary Table S8. Effects of dexmedetomidine (45 minutes) on the HCN activation time constant τ_{fast} at different concentration groups (absolute values). Wilcoxon-matched pairs signed rank test for statistical comparisons.

	**τ_{fast} [ms]**					
	**control**		**DEX**			
**DEX [µM]**	Median	IQR	Median	IQR	n	*p*
**1**	217.5	199.9–223.9	207.1	188.6–224.0	11	0.413
**10**	271.5	219.4–290.5	279.2	244.8–335.1	10	0.014
**100**	250.8	225.1–289.4	376.0	259.2–412.6	10	0.084

Supplementary Table S9. Effects of (45 minutes) on the HCN activation time constant τ_{slow} at different concentration groups (absolute values). Wilcoxon-matched pairs signed rank test for statistical comparisons.

	τ_{slow} [ms]					
	**control**		**DEX**			
**DEX [µM]**	Median	IQR	Median	IQR	n	*p*
**1**	1088	984.4–1329	1089	881.8–1190	11	0.765
**10**	1248	938.9–1482	1295	1041–1962	10	0.322
**100**	1227	1029–1608	941.8	735.4–1423	10	0.232

Supplementary Table S10. Effects of dexmedetomidine (45 minutes) on the voltage sag amplitude at different concentration groups (absolute values). Wilcoxon-matched pairs signed rank test for statistical comparisons.

	**voltage sag amplitude [mV]**					
	control		DEX			
**DEX [µM]**	Median	IQR	Median	IQR	n	*p*
**1**	85.07	71.68–89.16	84.90	74.20–87.41	11	0.577
**10**	91.29	78.67–96.41	87.33	80.76–95.77	10	0.492
**100**	71.22	65.70–90.50	85.72	68.19–93.15	10	0.014

Supplementary Table S11. Effects of dexmedetomidine (45 minutes) on the rebound burst delay at different concentration groups (absolute values). Wilcoxon-matched pairs signed rank test for statistical comparisons.

	**rebound burst delay [ms]**					
	**control**		**DEX**			
**DEX [µM]**	Median	IQR	Median	IQR	n	*p*
**1**	29.0	27.0–30.5	29.5	26.5–32.0	11	0.419
**10**	28.1	24.9–29.8	29.0	25.3–33.1	10	0.719
**100**	30.4	27.9–31.9	36.5	35.4–39.5	10	0.006

Supplementary Table S12. Effects of dexmedetomidine (45 minutes) on the number of action potentials during rebound at different concentration groups (absolute values). Wilcoxon-matched pairs signed rank test for statistical comparisons.

	**number of action potentials (n)**					
	**control**		**DEX**			
**DEX [µM]**	Median	IQR	Median	IQR	n	*p*
**1**	7.8	6.8–11.4	9.0	7.9–11.0	11	0.31
**10**	9.6	5.3–17.6	7.9	5.8–17.3	10	>0.999
**100**	10.5	7.8–17.3	4.0	2.9–8.3	10	0.002

Supplementary Table S13. Effects of sevoflurane (45 minutes) on the rebound burst duration at different concentration groups (absolute values). Wilcoxon-matched pairs signed rank test for statistical comparisons.

	**rebound burst duration [ms]**					
	**control**		**DEX**			
**DEX [µM]**	Median	IQR	Median	IQR	n	*p*
**1**	188.0	117.0–389.5	251.5	149.0–322.5	11	0.206
**10**	257.0	118.6–572.9	259.0	159.8–625.3	10	0.557
**100**	252.5	129.9–679.8	50.75	36.0–337.5	10	0.020
